# Adaptive attenuation of virulence in hypervirulent carbapenem-resistant *Klebsiella pneumoniae*

**DOI:** 10.1128/msystems.01363-23

**Published:** 2024-05-16

**Authors:** Gaoqin Teng, Meng Zhang, YingYing Fu, Xiaoqiang Yang, Yanhua Kang, Qiuying Qin, Ye Jin, Man Huang, Yongchang Xu

**Affiliations:** 1 Department of Immunology and Pathogen Biology, School of Basic Medical Sciences, Hangzhou Normal University, Hangzhou, China; 2 Key Laboratory of Multiple Organ Failure, Ministry of Education, Hangzhou, China; 3 Department of General Intensive Care Unit of the Second Affiliated Hospital, Zhejiang University School of Medicine, Hangzhou, China; University of California San Diego, La Jolla, California, USA

**Keywords:** hypervirulent carbapenem-resistant *K. pneumoniae*, ST11-KL64, *rmpA*, adaptive evolution, capsular polysaccharide

## Abstract

The emergence of nosocomial infections caused by hypervirulent and carbapenem-resistant *K. pneumoniae* (hv-CRKP) has become a significant public health challenge. The genetic traits of virulence and resistance plasmids in hv-CRKP have been extensively studied; however, research on the adaptive evolution strategies of clinical strains inside the host was scarce. This study aimed to understand the effects of antibiotic treatment on the phenotype and genotype characteristics of hv-CRKP. We investigated the evolution of hv-CRKP strains isolated from the same patient to elucidate the transition between hospital invasion and colonization. A comparative genomics analysis was performed to identify single nucleotide polymorphisms in the *rmpA* promoter. Subsequent validation through RNA-seq and gene deletion confirmed that distinct *rmpA* promoter sequences exert control over the mucoid phenotype. Additionally, biofilm experiments, cell adhesion assays, and animal infection models were conducted to illuminate the influence of *rmpA* promoter diversity on virulence changes. We demonstrated that the P12T and P11T promoters of *rmpA* possess strong activity, which leads to the evolution of CRKP into infectious and virulent strains. Meanwhile, the specific sequence of polyT motifs in the *rmpA* promoter led to a decrease in the lethality of hv-CRKP and enhanced cell adhesion and colonization. To summarize, the *rmpA* promoter of hv-CRKP is utilized to control capsule production, thereby modifying pathogenicity to better suit the host’s ecological environment.

**IMPORTANCE:**

The prevalence of hospital-acquired illness caused by hypervirulent carbapenem-resistant *Klebsiella pneumoniae* (hv-CRKP) is significant, leading to prolonged antibiotic treatment. However, there are few reports on the phenotypic changes of hv-CRKP in patients undergoing antibiotic treatment. We performed a comprehensive examination of the genetic evolutionary traits of hv-CRKP obtained from the same patient and observed variations in the promoter sequences of the virulence factor *rmpA*. The strong activity of the promoter sequences P11T and P12T enhances the consistent production of capsule polysaccharides, resulting in an invasive strain. Conversely, weak promoter activity of P9T and P10T is advantageous for exposing pili, hence improving bacterial cell attachment ability and facilitating bacterial colonization. This finding also explains the confusion of some clinical strains carrying wild-type *rmpA* but exhibiting a low mucoid phenotype. This adaptive alteration facilitates the dissemination of *K. pneumoniae* within the hospital setting.

## INTRODUCTION

*Klebsiella pneumoniae* (*K. pneumoniae*) is a commensal and opportunistic pathogen in humans that can cause community- and hospital-associated infections, leading to a variety of infectious diseases such as pneumonia, liver abscess, urinary tract infections, and bloodstream infections (1, 2). *K. pneumoniae* has evolved many tactics that facilitate the emergence and spread of bacterial antibiotic resistance and hypervirulence. Recently, with the epidemic of carbapenem-resistant and hypervirulent *K. pneumoniae*, nosocomial infections have become a major challenge in public health (3, 4). The hypervirulent and carbapenem-resistant *K. pneumoniae* strains reported globally can be categorized into two patterns: (i) KL1/KL2 hypervirulent *K. pneumoniae* (hvKP) obtained a carbapenem-resistant plasmid known as CR-hvKp (5) and (ii) carbapenem-resistant *K. pneumoniae* (CRKP) acquired a virulence plasmid, referred to as hv-CRKP (3, 6). In recent years, the prevalence of the KPC-producing strain ST11 hypervirulent carbapenem-resistant *K. pneumoniae* (ST11-hvCRKP) in intensive care units (ICUs) in China has become a serious public health crisis (4, 5).

The type and production of capsule polysaccharides are closely related to the virulence of *K. pneumoniae*. It is known that *K. pneumoniae* synthesizes more than 80 types of capsule polysaccharides, which are commonly referred to as K-antigens (7). Each type varies in the repeating polysaccharide unit of capsular polysaccharide (CPS). Meanwhile, *K. pneumoniae* capsule types exhibit significant variability in virulence characteristics (8). The hypervirulent KL1 and KL2 capsular serotypes are known to cause serious community-acquired infections such as pyogenic liver abscesses (9, 10). Notably, the acquisition of virulence plasmids such as pLVPK was the main factor contributing to the hypervirulent phenotype of *K. pneumoniae* (11). The virulence plasmid carried many virulence determinants, including an iron acquisition system, the *iucABCD* cluster, the *iroBCDN* siderophore operon, and the ferric aerobactin acceptor-encoding gene *iutA* (12, 13). Most importantly, it harbored two regulatory genes (*rmpA* and *rmpA2*) that contribute to the hypermucoid phenotype (14, 15). The hypermucoid phenotype of hvKP, which is caused by the chain length or the overexpression of CPS, is generally regarded as a crucial hallmark of hypervirulence (16, 17). CPS is known for its ability to prevent host phagocytic clearance to facilitate immune evasion and survival of encapsulated bacteria.

Approximately 26 structural genes make up the CPS cluster, which is located on the chromosome and encodes several enzymes, including transporters, ligases, and glycosyltransferases, such as Wza, Wzc, Wzi, WcaJ, and WbaP (18, 19). In addition, mutations in several capsule biosynthesis genes in the CPS could result in *K. pneumoniae* changing its pathogenicity (20). The *wcaJ* produces a glycosyltransferase that is essential for the synthesis of polysaccharides. Inactivation of the *wcaJ* gene via the insertion sequence (IS) in ST23-KL1 hvKP was shown to be associated with a low fitness cost and a high conjugation capacity (21, 22). The role of *wzc* in contributing to hypercapsule production is also well characterized in *K. pneumoniae* ST258, which leads to enhanced phagocytosis resistance, dissemination, and virulence. Single-residue substitutions in genes (*wzc*, *wcaJ,* and *wbaP*) regulate the virulence and persistence of *K. pneumoniae* through two opposite evolutionary mechanisms (21–23). Moreover, many transcription factors, including *rmpA*, *rmpA2*, *rmpC,* and *rmpD,* are often found in virulence plasmids and upregulate the synthesis of CPS (14, 15, 24, 25). Mutations in the two genes *rmpA* and *rmpA2* can cause hvKp to bereft the hypermucoid phenotype (for which the string test is negative). Interestingly, some clinical isolates bore wild-type *rmpA* but displayed a hypovirulent phenotype (26, 27). This enigmatic evolutionary strategy of *K. pneumoniae* may be related to its fitness in the host, which requires further investigation.

In this study, we described the separation of two unique ST11-KL64 CRKP mucoid phenotypes from a patient and examined the causes of these phenotypic variations. Through gene deletion, cell adhesion assays, whole-genome sequencing (WGS), transcriptome sequencing, and animal infection models, we uncovered the variety of *rmpA* promoter activities and their capacity to control mucoid characteristics so that some isolates can more effectively adapt to the host environment. Our findings illuminated that the diversity of *rmpA* promoters plays a crucial role in the evolution of the pathogenicity of within-host adaptation in ST11-KL64 CRKP strains and provided a novel perspective for monitoring hv-CRKP in the clinical setting.

## RESULTS

### Phenotypes of pathogenicity of *Klebsiella pneumoniae* isolated from the clinic

**Fig 1:**
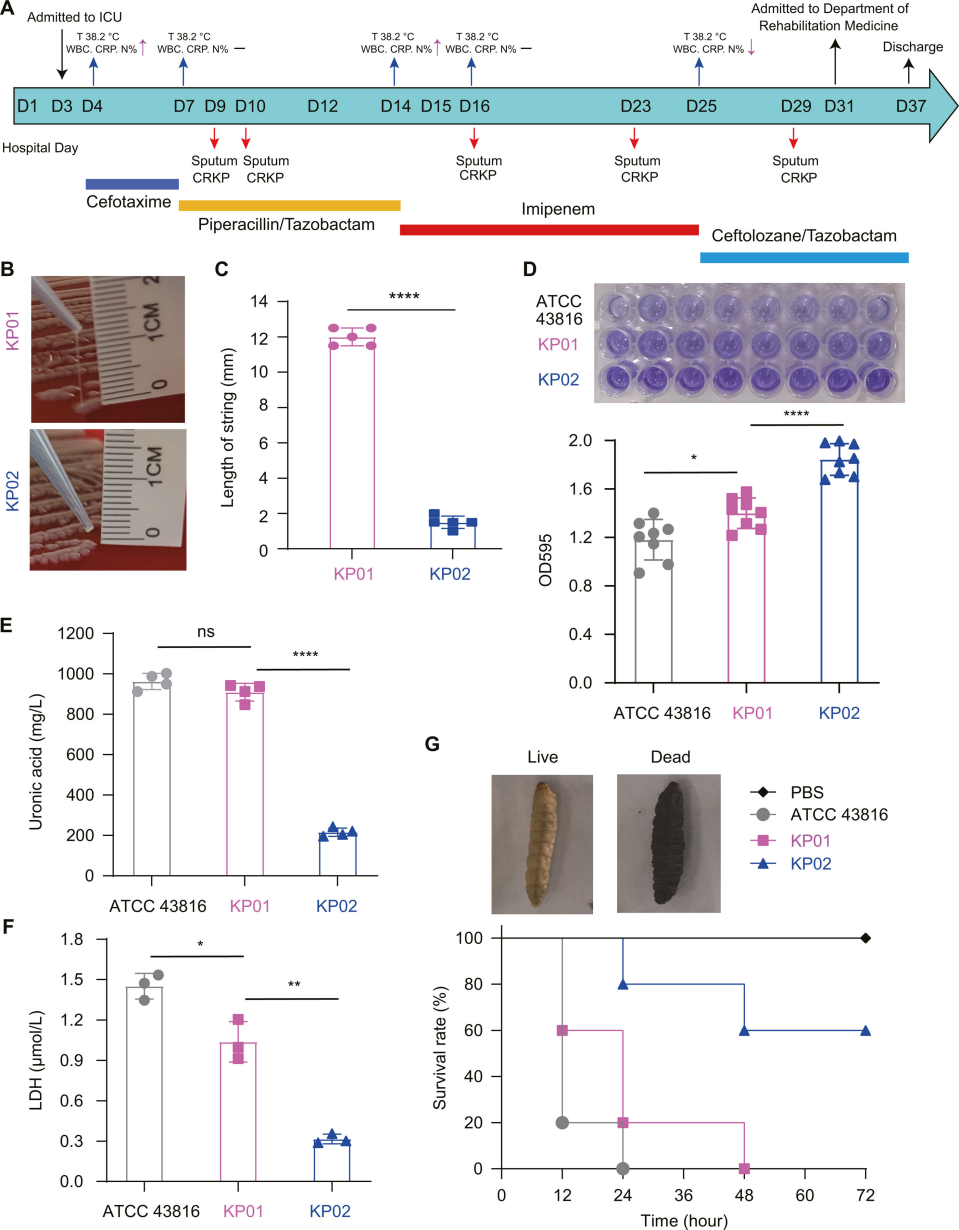
The virulence phenotypes and levels of *K. pneumoniae* KP01 and KP02. (**A**) Medical history of the patient. The different colored bars represent the different antibiotics used during treatment: blue bar (cefotaxime), yellow bar (piperacillin/tazobactam), red bar (imipenem), and pale blue bar (ceftolozane/tazobactam). The colored arrows correspond to different phenotypes. The red arrows represent the isolated *K. pneumoniae* strains. The blue arrows correspond to the patient’s physiologic status, such as temperature and immune marker data. The black arrows represent key points in the patient’s treatment. (**B and C**) Determination of the mucoid phenotypes of KP01 and KP02 by string tests on blood agar plates. (**D**) Determination of the biofilm production of the isolated strains. The *K. pneumoniae* ATCC 43816 was used as the control strain. (**E**) Capsule biomass of the strains quantified by uronic acid assay. Hypermucoid srains produce more capsules than normal mucoid isolates. (**F**) The lactate dehydrogenase (LDH) activity in human A549 lung epithelial cells was treated with three different strains. (**G**) The virulence of the isolates was assessed by *G. mellonella* infection. A one-way ANOVA and Tukey’s post-hoc test were performed for uronic acid measurement, biofilm formation, and LDH production. A log-rank (Mantel–Cox) test was performed for the survival curves. ***P < 0.001, ****P < 0.0001, ns, not significant.

In November 2018, a patient was taken to the hospital because of a serious spinal injury. Subsequently, on the third day, the patient was transferred to the ICU. In response to the observed elevation in the patient’s body temperature, a preventive regimen involving the administration of cefotaxime was initiated. In our study, *K. pneumoniae* was observed on multiple occasions, specifically on day 9, day 10, day 16, day 23, and day 29. Simultaneously, there was a transition to a more potent class of antibiotics. (Fig. 1A). Antibiotic sensitivity tests revealed that all five *K. pneumoniae* strains exhibited an identical spectrum of antibiotic resistance, specifically demonstrating resistance to carbapenem (Table 1). Initially, it was hypothesized that the five strains were identical clones. However, further experimentation utilizing the string test revealed that the first three strains showed positive results and were designated KP01, while the subsequent two strains exhibited negative results and were named KP02. It is postulated that the first three strains were derived from the same genetic lineage, whereas the last two strains were genetically identical. The application of XbaI-pulsed-field gel electrophoresis (PFGE) revealed that KP01 and KP02 had identical genetic contexts. Moreover, via PCR, we ascertained that both strains harbor the drug resistance gene *bla*_KPC_, as well as the virulence genes *rmpA*, *rmpA2*, *iucA*, *iroN*, and *iutA*. Notably, these genes are typically located on the plasmid (Fig. S1A and B). Nevertheless, despite their essentially identical genetic backgrounds, the two CRKP strains exhibit contrasting mucoid characteristics. Specifically, KP01 displays a string length of approximately 12 mm, whereas KP02 is only 2 mm (Fig. 1B and C). Consequently, we proceeded to conduct a comprehensive investigation of these two CRKP strains.

**TABLE 1:** Comparing the antibiotic resistance profiles of KP01 and KP02

Antibiotics	KP01		KP02	
	MIC	Sensitivity	MIC	Sensitivity
Tigecycline	4	I	4	I
Imipenem (IPM)	≥16	R	≥16	R
Meropenem (MEM)	≥16	R	≥16	R
Colistin	≤0.5	S	≤0.5	S
Piperacillin/tazobactam (TZP)	≥128	R	≥128	R
Amikacin (AMK)	≥64	R	≥64	R
Tobramycin (TOB)	≥16	R	≥16	R
Cefoperazone/sulbactam (SFP)	≥64	R	≥64	R
Aztreonam (ATM)	≥64	R	≥64	R
Ciprofloxacin (CIP)	≥4	R	≥4	R
Levofloxacin (LVX)	≥8	R	≥8	R
Ceftazidime (CAZ)	≥64	R	≥64	R
Minocycline (MNO)	≥16	R	≥16	R
Doxycycline	8	I	8	I
Cefepime (FEP)	≥31	R	≥31	R
Puromyn	≤20	S	≤20	S

**Fig 2:**
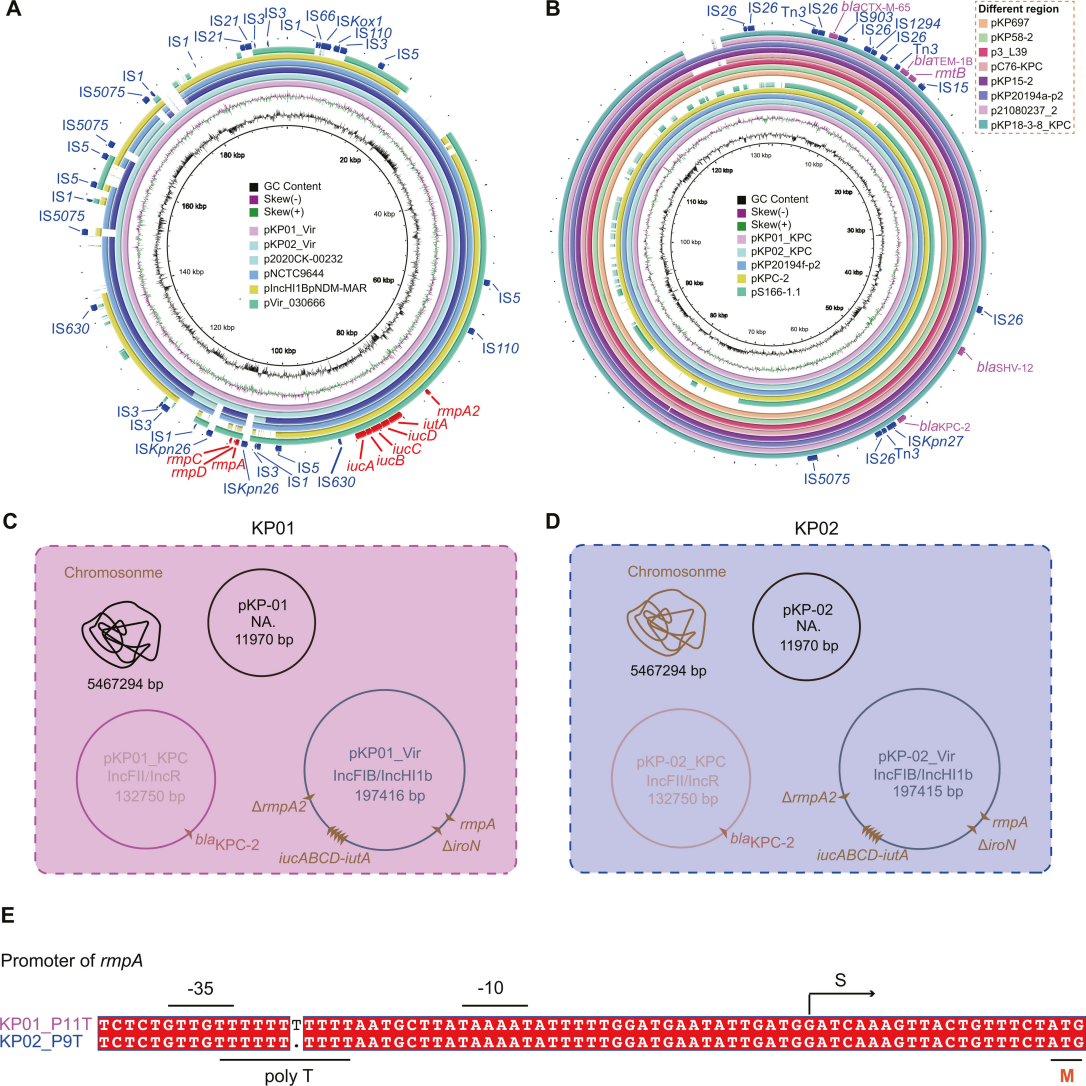
Comparative genomic analysis of genetic context between distinct phenotypes of two strains KP01 and KP02. (**A**) Circular analysis of the IncHI1B virulence plasmids pKP01-Vir, pKP02-Vir, p2020CK-00232, pNCTC9644, pIncH1BpNDM-MAR, and pVir_030666 in the NCBI database using BRIG. (**B**) Alignment of the *bla*KPC-2-bearing plasmid pKP01-KPC with pKP02-KPC, pKP697, pKP58-2, p3_L39, pC76_KPC, pKP15-2, pKP20194a-p2, p21080237-2, and pKP18-3-8 using BRIG. (**C and D**) Scheme for two strains of *K. pneumoniae* ST11-KL64, named KP01 and KP02. The KP01 strain contains three different plasmids. Specifically, they contained (i) the IncHI1B/IncHI1b plasmid pKP01_Vir (~197.5 kb), (ii) the pKP01_KPC plasmid (~132.7 kb), and (iii) the new typable small plasmid pKP01_3 (~11.9 kb). In addition to five antibiotic resistance genes (*bla*CTX-M-65, *bla*TEM-1B, *rmtB*, *bla*SHV-12, and *bla*KPC-2) located on pKP01_KPC and pKP02_KPC, the virulence plasmid included a series of virulence-related elements (Δ*rmpA2*, *iucABCD-iutA*, *rmpA*, and Δ*iroN*). (**E**) Sequence comparison analysis of the *rmpA* promoter. The promoter region of *rmpA* contains the polyT motif. P10T stands for the *rmpA* promoter containing 10T tract, and P11T refers to the 11T tract containing the *rmpA* promoter. The known transcription start location is indicated by the letter “S” at the top of an arrow. The regions designated as “−10” and “−35” are highlighted.

To investigate the differences in string-test traits between KP01 and KP02, we conducted whole-genome sequencing and determined that both strains belonged to the ST11-KL64 lineage, which is widespread in China. In our study, a systematic comparison was conducted to assess the virulence of KP01 and KP02. The data indicated that KP02 displayed a marginally superior growth rate compared to KP01 during the duration of the growth curve. Furthermore, the mucoviscosity sedimentation assay revealed that KP01 had greater mucoviscosity than KP02 (Fig. S1C). The results of the quantitative biofilm assays demonstrated that KP01 exhibited a lower level of biofilm production than KP02. However, KP01 displayed a biofilm production level similar to that of the hypervirulent ST493-KL2 type *K. pneumoniae* ATCC 43816 which was used as the control (Fig. 1D). As the string test showed that KP01 was positive and KP02 was negative, we further performed quantitative assays for the capsules. Uronic acid (UA) is an essential constituent and a biomarker of the capsule. UA was measured in KP01 and KP02 from late-log cultures grown in LB medium. KP01 has a concentration of approximately 909.5 mg/L, which closely resembles that of hypervirulent *K. pneumoniae* ATCC 43816 at 962.95 mg/L. In contrast, KP02 had a concentration of approximately 216.3 mg/L. The KP02 strain exhibited a significantly lower level of UA production than the KP01 strain, with a reduction of approximately 4.5-fold (Fig. 2E).

We further assessed the pathogenicity of two ST11-KL64 KP01 and KP02 in lung epithelial cell A549 and *G. mellonella* larval infection model. The more invasive strains were to the cell, the more lactate dehydrogenase (LDH) was released (28). KP01 significantly increased LDH activity in human lung epithelial cells compared to the KP02 strain (1.037 µmol/L vs 0.3157 µmol/L). Compared to the control strain KL2 hypervirulent *K. pneumoniae* ATCC 43816, the ST-KL64 KP01 strain produced lower levels of LDH (1.451 µmol/L vs 1.037 µmol/L) (Fig. 1F). We also verified the virulence of two strains, KP01 and KP02, by infecting *G. mellonella*. By injecting 10^6^ CFU bacteria into *G. mellonella*, the survival status of different strains within 72 h was compared, and phosphate-buffered saline (PBS) was injected as a control. We found that all *G. mellonella* infected with ATCC 43816 died at 24 h, and all *G. mellonella* infected with KP01 died at 48 h. However, 40% of those infected with KP02 died within 48 h, and no deaths occurred within 72 h (Fig. 1G).

### Comparative genetic context of the ST11-KL64 CRKP strains

This intriguing observation stems from the shared features of both strains, encompassing their affiliation with the ST11-KL64 genotype and possession of both virulence and resistance plasmids (containing *rmpA* and *bla*KPC, respectively). However, despite these similarities, they displayed significantly different levels of pathogenicity. Therefore, based on WGS results, KP01 and KP02 both contained three plasmids, pKP01/pKP02, pKP01_KPC/pKP02_KPC, and pKP01_Vir/pKP02_Vir, with sizes of 11.9 kb, 132.7 kb, and 197.5 kb, respectively (Fig. 2A and B). The virulence plasmids pKP01_Vir and pKP02_Vir are almost identical, with only one base pair difference. On the pKP02_Vir plasmid, there is a T base deletion in the *rmpA* promoter region (Fig. 2E). The virulence factors included *rmpA*DC, *rmpA*2 (frameshift mutation), *iutA,* and *iucABCD* clusters (Fig. S2). The virulence plasmid was highly similar to the *K. pneumoniae* plasmids derived from several hospitals in Hangzhou, such as p2020CK-00232, pNCTC9644, pIncH1BpNDM-MAR, and pVir_030666, with >99% query coverage and 99% identity. These plasmids belonged to the IncH1B type and may be an important tool to promote the spread of the *rmpA* gene among hospitals. The *rmpA* gene was predicted to be located on a genetic island mediated by IS*Kpn26* (Fig. 2C and D). The resistance plasmids pKP01_KPC and pKP02_KPC are completely identical and possess multiple resistance genes, including *bla*KPC-2, *bla*CTX-M-65, *bla*TEM-1B, and *rmtB* (Table 2). This multidrug-resistant plasmid is frequently encountered in *K. pneumoniae* strains originating from hospital settings. Notably, pKP01-KPC exhibited homology to plasmids isolated from various environmental sources, including those found at the nurses’ station (pKP20194f-p2, CP054722), elephant feces (pKPC-2, CP130265), and pangolins (pS166-1.1, CP063946). This finding suggests a potential for the dissemination of this plasmid within environmental reservoirs and across diverse animal-associated bacterial populations (Fig. 2B). We also performed a comparison of the chromosomes between KP01 and KP02, identifying only 10 SNP variations between them (Table S1), and these mutation sites are all unrelated to bacterial virulence. We hypothesized that SNP changes in the virulence plasmid may lead to divergent virulence phenotypes.

**TABLE 2:** A group of resistant and virulent plasmids with sequenced genomes in this work

Plasmids	Plasmid types	Size (bp)	Resistance determinants
pKP01_KPC	IncFII/IncR	132750	*rmtB*, *bla*_KPC-2_, *bla*_SHV-12_, *bla*_TEM-1B_, *bla*_CTX-M-65_
pKP01_Vir	IncHI1B	197516	*rmpA*, Δ*rmpA2*, *iucABCD*, *iutA*, *iroN*
pKP01_3	NA	11970	NA
pKP02_KPC	IncFII/IncR	132750	*rmtB*, *bla*_KPC-2_, *bla*_SHV-12_, *bla*_TEM-1B_, *bla*_CTX-M-65_
pKP02_Vir	IncHI1B	197515	*rmpA*, Δ*rmpA2*, *iucABCD*, *iutA*, *iroN*
pKP02_3	NA	11970	NA

### RNA-seq analysis of differentially expressed genes between KP01 and KP02

**Fig 3:**
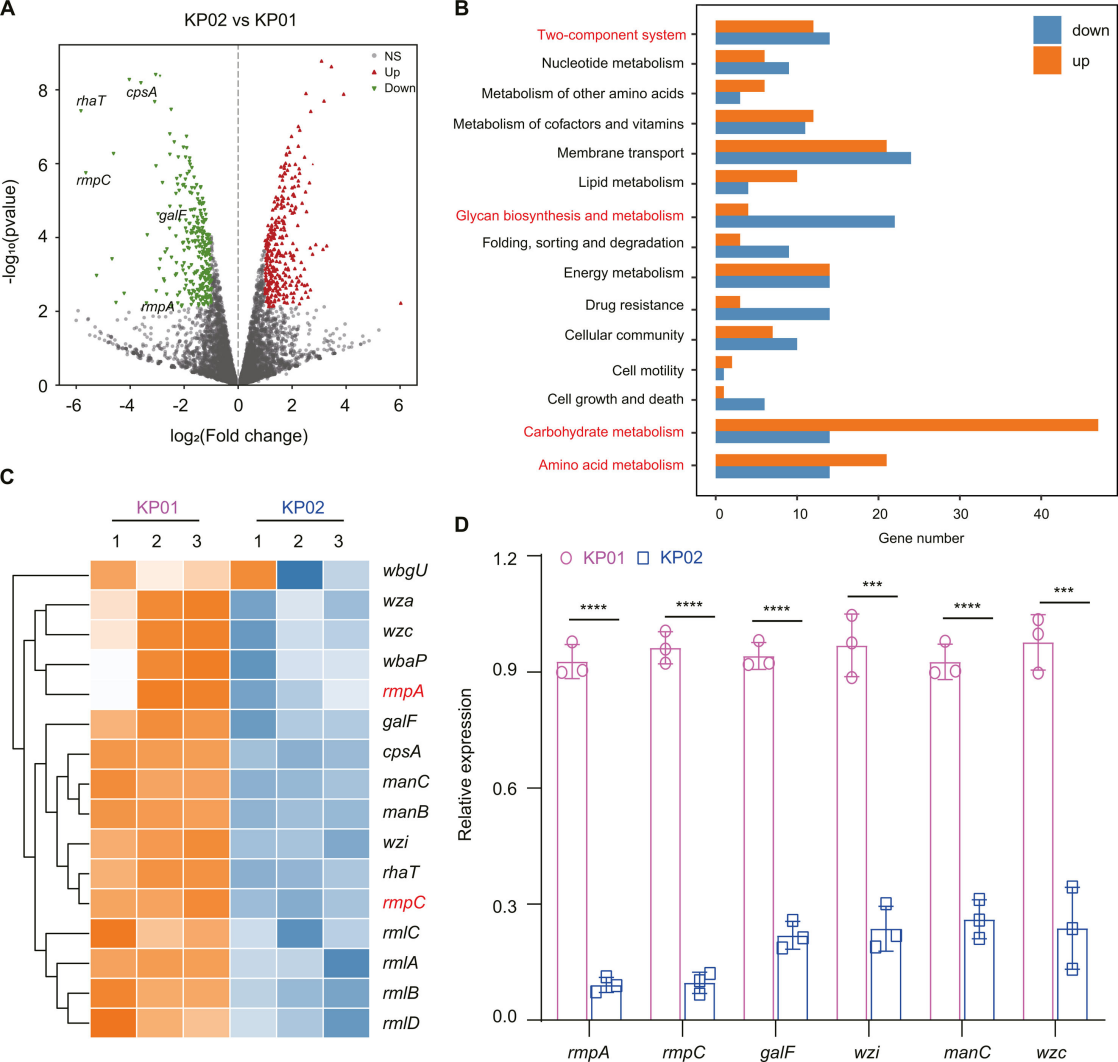
The transcriptome analysis of KP01 and KP02. (**A**) Volcano plot of the differential gene expression levels between KP01 and KP02. Each dot represents a single gene. Red dots represent genes whose abundance is significantly upregulated, green dots downregulated, and black dots represent no significant change. (**B**) The number of upregulated (orange bar) and downregulated (blue bar) DEGs related to metabolism. (**C**) Heatmap of differentially expressed genes related to the capsular synthesis cluster. The *z* score (1.5 to −2.0) indicates whether the genes were upregulated (orange) or downregulated (blue). (**D**) RT-qPCR analysis of *cps* gene expression. The data are presented as the mean  ±  SD (*n*  =  3). Normalize RNA transcripts relative to wild-type controls using the 2^−ΔΔCt^ threshold cycling method, with *rpoB* as the internal reference control.

To understand the differences in pathogenicity between KP01 and KP02, the gene expression profiles of the two strains were analyzed via RNA-seq. A total of 587 differentially expressed genes (DEGs) were identified between KP01 and KP02, of which 289 genes were downregulated and 298 were upregulated (Tables S2 and S3). Among the downregulated genes, there were significant changes in the genes involved in the capsule synthesis cluster (CPS) and regulation, such as structural genes (*galF*, *rhaT,* and *cpsA*) and regulatory factors (*rmpA* and *rmpC*) (Fig. 3A). We also found by KEGG clustering analysis that most of the genes involved in polysaccharide anabolism were downregulated in KP02 compared to KP01, while the expression of genes involved in amino acid metabolism and carbohydrate metabolism was significantly upregulated, which may be an important factor in the faster growth of KP02 compared to KP01 (Fig. 3A and B). Notably, almost all the CPS cluster genes (16 of 24 genes) exhibited a 2-fold downregulation in the transcriptome (Fig. 3C), suggesting that the negative string test observed in KP02 could be attributed to inhibited capsule synthesis. Among the DEGs involved in capsule synthesis, six downregulated genes (*rmpA*, *rmpC*, *galF*, *wzi*, *manC,* and *wzc*) were selected and validated via RT‒qPCR; these genes exhibited consistent gene expression changes (Fig. 3C and D), revealing that the quality of the transcriptome data was good and that genes associated with capsular synthesis, indeed, declined. We compared the gene clusters for CPS synthesis in KP01 and KP02 and found that the sequences were identical. Therefore, our results indicated that the transcription level of the capsule synthesis gene cluster of KP02 isolate was significantly inhibited.

### Effect of *rmpA* promoter mutation on the virulence of *K. pneumoniae*

Why did the mucoid regulatory factors *rmpA* and *rmpC* of KP01 and KP02 show different expression levels? We hypothesized that this discrepancy might arise from mutations within the *rmpA* promoter region (Fig. 2E). The CRISPR/Cas9 system was adopted for deleting *rmpA* from the virulence plasmid localized on the two strains KP01 and KP02 (Fig. S3A). Next, we verified the positive colonies of the deletion mutant by PCR and Sanger sequencing (Fig. S3B). Moreover, we constructed strains curing the virulence plasmid pKP01_vir and pKP02_vir named KP01pc and KP02pc, respectively; Constructed the P11T-*rmpA* and P10T-*rmpA* vectors on pSGKP-Rif; and backfilled KP01Δ*rmpA* and KP02Δ*rmpA*, respectively, to obtain the strains of P11T *rmpA*/KP01Δ*rmpA* and P10T *rmpA*/KP01Δ*rmpA*, as well as P11T *rmpA*/KP02Δ*rmpA* and P10T *rmpA*/KP02Δ*rmpA* strains.

**Fig 4:**
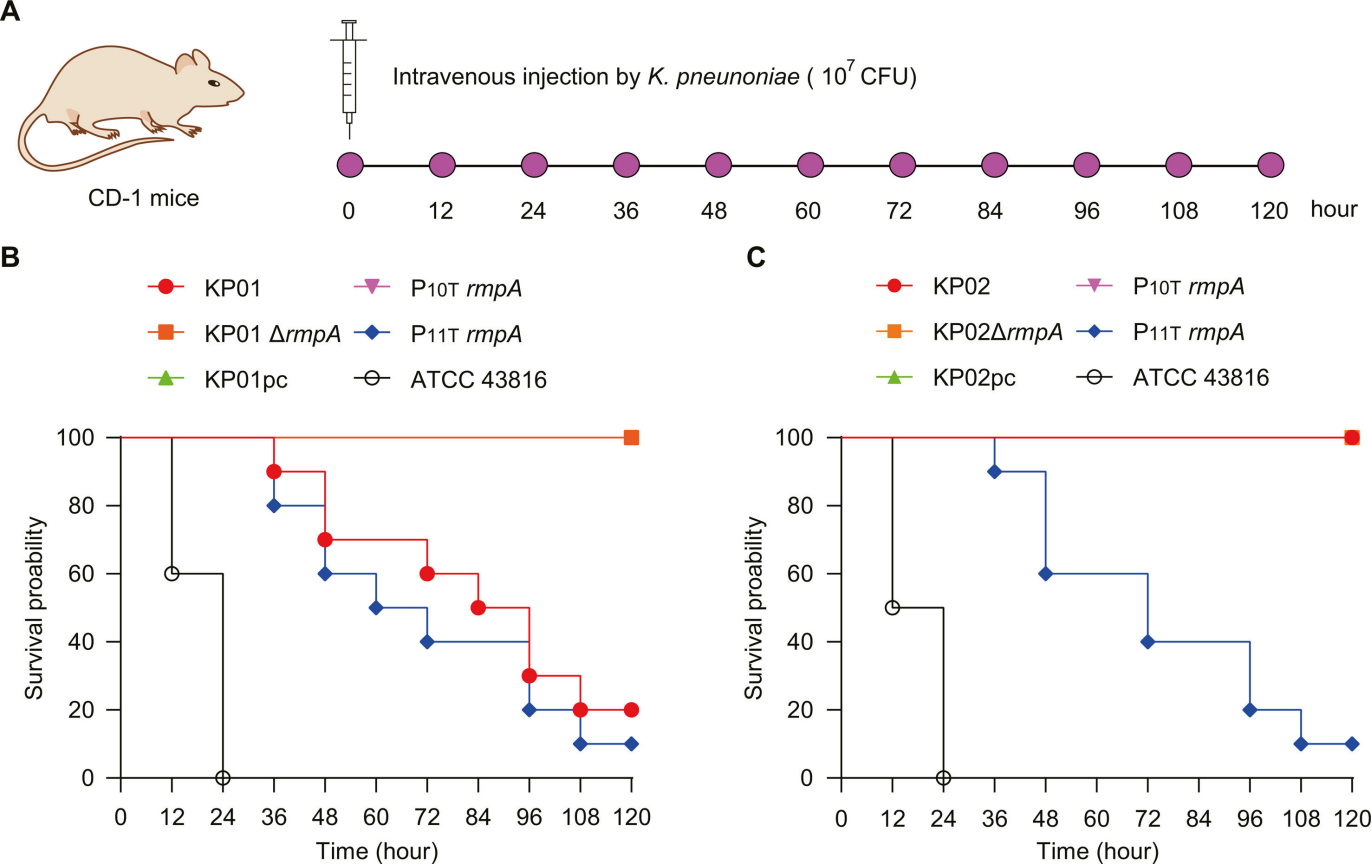
The effects of the promoter of *rmpA* on the virulence of KP01 and KP02 were evaluated in mice infection model. (**A**) Diagram of the mouse infection with *K. pneumoniae* strains. (**B and C**) The survival curves of mice infected with the *rmpA*-bearing KP01, KP02, and its different derivatives 6 groups of CD-1 mice (10 per group) were treated via mouse tail intravenous injection. Three independent experiments were conducted. The hypervirulent *K. pneumoniae* ATCC 43816 is the control strain (labeled as gray).

The virulence of the different strains was tested in a mouse infection model (Fig. 4A), the results of which showed that KP01 exhibited higher virulence levels than did the KP02 strain and that both strains exhibited markedly lower virulence levels than did the control strain ATCC 43816 (Fig. 4B and C). A mouse sepsis model demonstrated that at an inoculum of 1 × 10^7^ CFU, KP01 led to a high mortality rate, while KP01Δ*rmpA*, KP01pc, and P10T-*rmpA* were nonlethal under similar conditions at 120 h post-infection. Moreover, the fatality rate of the P11T-*rmpA*/KP01Δ*rmpA* strain was greater than that of KP02 (Fig. 4B). Moreover, the P11T-*rmpA*/KP02Δ*rmpA* strain was highly lethal to mice compared to the other strains KP02, KP02Δ*rmpA*, KP02pc, and P10T-*rmpA*/KP02Δ*rmpA* (Fig. 4C). These results suggested that the *rmpA* gene is critical for the pathogenicity of ST11-KL64 CRKP. However, mutations in the *rmpA* promoter region alter the production of capsular polysaccharides, which, in turn, affects the virulence of the bacterium. We found that changes in single-nucleotide polymorphisms (SNPs) (one T base deletion) in the *rmpA* promoter region of KP02 significantly reduced its virulence compared to that of KP01.

Typical syndromes in infected mouse tissues were observed through histopathological analysis. A histological examination of the lung, liver, and kidney tissues obtained from a cohort of infected CD-1 mice was conducted using hematoxylin and eosin (HE) staining. The control group in this study was treated with PBS (Fig. S4A). Pathology revealed characteristic manifestations of hvKP infection syndrome, such as liver inflammatory cell infiltration, congestion, and liver abscess. The introduction of KP01 resulted in the manifestation of significant hepatic and renal disorders (Fig. S4B). In contrast, the mice that received KP02 (P10T-*rmpA*) did not exhibit notable tissue damage in their liver or kidneys, consistent with their characteristic diminished virulence (Fig. S4C). In essence, active PT11-*rmpA* regulation plays a pivotal role in determining the virulence attributes in ST11-KL64 CRKP clones.

**Fig 5:**
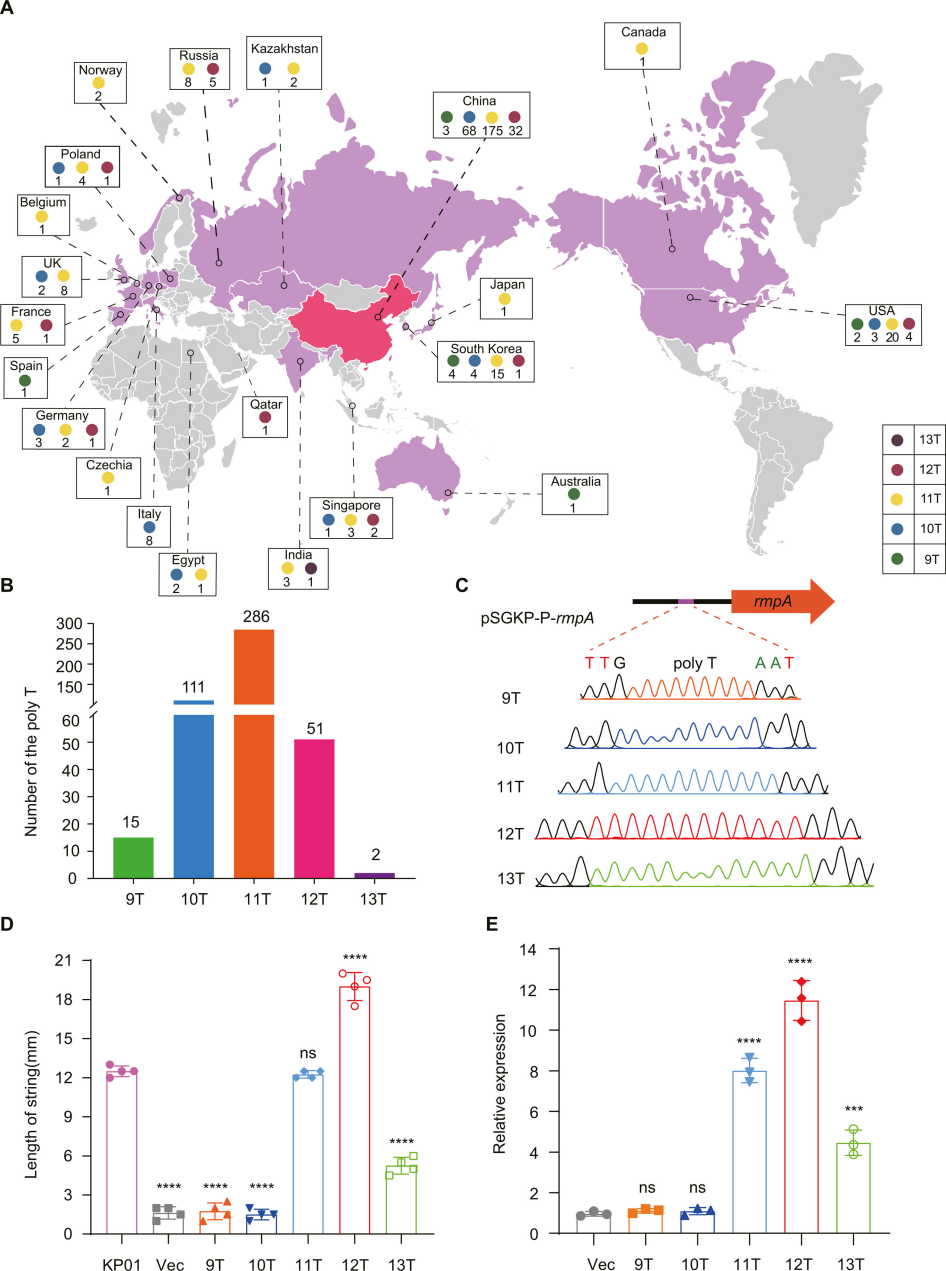
The nucleotide polymorphisms in the *rmpA* promoter region influence the pathogenicity of *Klebsiella pneumonia.* (**A**) The diversity of the promoter region of *rmpA* in *Klebsiella pneumoniae* was investigated worldwide. The variety of the *rmpA* promoter was determined via a BLAST search of the NCBI database. The different *rmpA* promoters were divided into five sequences (P9T to P13T). (**B**) The number of five different sequences of *rmpA* promoters in *K. pneumoniae*. (**C**) Design of *rmpA* vectors containing five different types of promoters. (**D**) Assess the activity of five different *rmpA* promoters in KP01Δ*rmpA* strain using a string test. (**E**) RT‒qPCR analysis of the effects of five different promoters on the transcript level of *rmpA.* The map was obtained from free amcharts website.

To determine whether there is diversity in the promoter region of *rmpA* in *K. pneumoniae*, we investigated the NCBI database using the promoter of *rmpA* (named P11T) from KP01 as a template. Globally, in 21 countries, 5 promoter sequences were found, namely, P9T, P10T, P11T, P12T, and P13T; P11T accounted for the majority of the strains (286); and the others were observed 15, 111, 51, and 2 times (Fig. 5A and B) (Table S4), respectively. These findings suggested that *K. pneumoniae* may have undergone some selection pressures, resulting in the development of strategies to adapt to the external environment through mutations within the *rmpA* promoter region.

To verify the activity of the five different promoter sequences, we artificially constructed and inserted *rmpA* with five sequences of promoters into the pSGKP plasmid to complement the KP01Δ*rmpA* strain. Through the string test, it was found that the string lengths of the promoters with P12T and P11T were approximately 19 cm and 12.25 cm, respectively; those with P9T and P10T were almost the same, approximately 1.5 cm; and those with 13T were 5 cm (Fig. 5D), indicating that the promoters of P12T and P11T were the most vigorous. Furthermore, RT-qPCR demonstrated that the expression level of *rmpA* was greatest for the P11T and P12T promoters, especially for the P12T promoter, in which the expression was approximately sevenfold greater than that of P9T and P10T (Fig. 5E). These results confirm our hypothesis that *K. pneumoniae* regulates its pathogenicity by altering the activity of the *rmpA* promoter. This also explains why some *K. pneumoniae* strains, such as the KP02 strain, harbor a virulence plasmid harboring complete *rmpA* genes but do not exhibit a hypervirulent phenotype. However, why do some *K. pneumoniae* isolates exhibit hypovirulence in the host? For example, does ST11-KL64 Hv-CRKP KP01 be detected on the 9th day of infection but transformed into Lv-CRKP KP02 on the 23rd day (Fig. 1A)? Therefore, we conducted the next step of the experiments.

### Colonization of KP01 and KP02 in the lungs of mice

**Fig 6:**
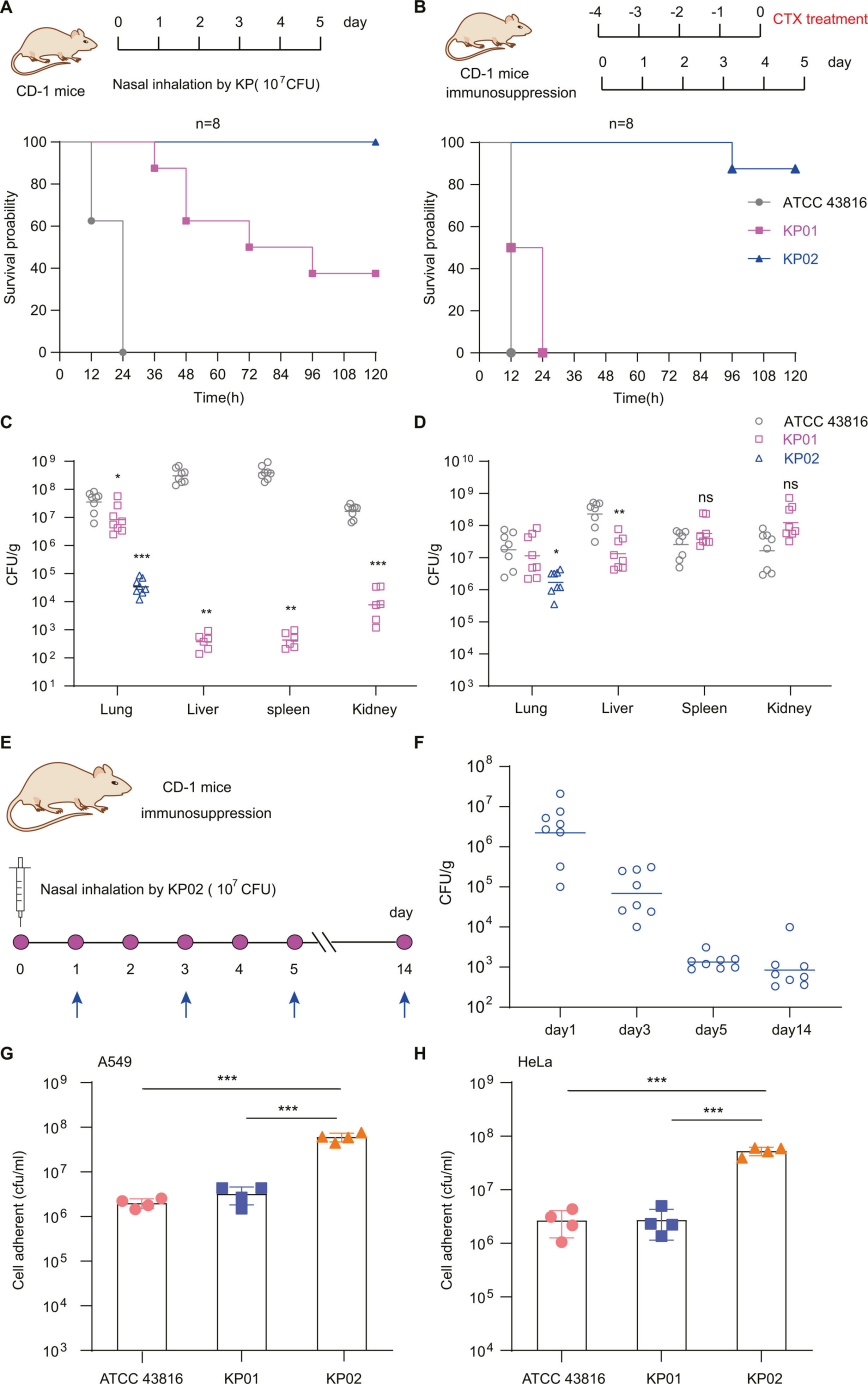
The host fitness of the mutations in the promoter region of *rmpA.* (A) Comparison of the virulence of KP01 and KP02 in mice via nasal inhalation. (**B**) An immunocompromised mouse model was used to assess the virulence of two strains, KP01 and KP02. (**C and D**) Bacterial loads in the lungs, liver, spleen, and kidneys following respiratory tract infection with KP01 (*n* = 8), KP02 (*n* = 8), or KP (*n* = 8). *K. pneumoniae* ATCC 43816 was used as the control strain. Mice were infected with KP01 (magenta) or KP02 (blue) bacteria via nasal inhalation injection (10^7^ CFU). Three independent experiments were conducted. (**E**) A schematic diagram showing the colonization of immunosuppressed mice by KP02 in the lungs. (**F**) Bacterial load of KP02 in the lungs of immunocompromised mice over time (*n* = 8); the line in the box shows the median. Three independent experiments were conducted. (**G and H**) Assessment of the cell adhesion ability of KP01 and KP02 using the A547 and HeLa cell lines.

We designed two mouse models using CD-1 mice (6 weeks): one with normal immunity and the other with immunosuppressive agents treated with cyclophosphamide (Fig. 6A and B). In normal mice, KP01 still had high virulence, with a survival rate of 40% within 120 h, and KP02 was not lethal to mice. However, the virulence of the control strain ATCC 43816 was greatest, with a mortality rate of 100% within 24 h (Fig. 6A). In the immunosuppressed mouse group, KP01 exhibited a stronger hypervirulent phenotype, killing 100% of the mice within 24 h, whereas the control strain ATCC 43816 killed all the mice within 12 h, and the KP02 strain killed only one mouse (repeated 3 times, 1 mouse died) (Fig. 6B). These results indicate that KP02 has low virulence even in immunodeficient mice.

We further investigated systemic *K. pneumoniae* infection *in vivo*. Mice typically undergo acute systemic *K. pneumoniae* infection and exhibit high bacterial burdens in tissues, such as the spleen, liver, kidney, and lungs. We selected mice infected for 24 h in two different groups of infection models and observed the bacterial loads in the lungs, liver, spleen, and kidney (Fig. 6C and D). The mice in the routine group, the hypovirulent *K. pneumoniae* KP02, was observed only in the lungs and was not detected in any other tissues. The hypervirulent KP01 strain of ST11-KL64 was loaded in all four organs, and the bacterial load in the lungs was approximately 10^7^ CFU/g, which was significantly greater than that in KP02 (10^4^ CFU/g); however, the load was significantly lower in all three organs than that in the control ATCC 43816 KL2 hypervirulent strain (Fig. 6C). In immunosuppressed mice, the KP02 isolate was still detected only in the lungs and not in the other three tissues, but the bacterial load was already greater than that the mice in the normal group. In contrast, KP01 and ATCC 43816 infections had high loads in all four organs, between 10^7^ and 10^8^ CFU/g, with no significant difference between the two strains (Fig. 6D).

The above results revealed that KL2-type *K. pneumoniae* had strong lethality against both groups of mice, and hypervirulent ST11-KL64 KP01 had a more significant infestation ability against immunosuppressed mice than against normal mice. Under nasal inhalation conditions, KP02 was only detected in lung tissue, indicating that KP02 may have colonized the respiratory tract of mice and will not cause fatal infections. Therefore, we extended the observation time to 14 days (Fig. 6E). The number of KP02 cells gradually decreased over time but stabilized on the 5th day. Until the 14th day, KP02 was still detected in the lungs of the mice (Fig. 6F), indicating that KP02 may adapt to the environment of the lungs. The results of cell adhesion assays also suggested that, compared with KP01, KP02 has a notably greater capacity for adhering to the surface of cells (Fig. 6F and G). Overall, whereas KP02 has limited pathogenicity, it has an increased capacity for cellular adhesion and improved persistence inside the host.

## DISCUSSION

The worldwide notorious pathogen *K. pneumoniae* is typically recognized as a rising source of infectious diseases in both hospitals and communities (29). Some clinical strains of *K. pneumoniae* exhibit adaptive mechanisms to survive effectively and thrive in different settings and hosts due to a diverse range of genetic evolutionary techniques. The adaptive evolution mechanism of *K. pneumoniae* is closely associated with mutations occurring in genes responsible for capsular polysaccharide synthesis (26, 29–31). The study conducted by Ernst et al. demonstrated that mutations in the *wzc* responsible for capsule biosynthesis result in excessive capsule production. This overproduction enhanced resistance against phagocytosis, promoted the spread of the pathogen, and increased mortality (21). Similarly, the Wzc (S682N) mutation induced a hypermucoid phenotype, which imposed a fitness cost, diminished biofilm formation, and reduced epithelial cell adherence, yet augmented resistance to macrophage phagocytosis and virulence (22). Under some selection pressures, strains with mutations in the *cps* locus of *K. pneumoniae* and resulting in altered chain length of capsule polysaccharides are selected. Saroj et al. discovered that certain mutations in *wzc* were adequate to prevent urine-mediated mucoid suppression. Even in urine, these Wzc variations result in the constitutive synthesis of more homogeneous capsular polysaccharide chains and enhanced release of the capsule from the cell surface (31). Disruption of *wcaJ* led to a nonmucoid phenotype, which promoted *in vitro* biofilm formation and epithelial cell adherence but reduced resistance to macrophage phagocytosis and virulence (22, 23). SNP mutations in capsule synthesis genes mostly affected the chain length of capsule polysaccharides, and our research results found that the KP02 isolate of *K. pneumoniae* indirectly reduces the production of capsule polysaccharides to enhance its host adaptability. In addition, Liu’s recent work found that some clinical isolates of *K. pneumoniae* exhibit the rdar phenotype, which is due to the BcsA (G579D) mutation increasing the production of cellulose, thereby enhancing its retention in environmental materials, while reducing the virulence to mice. These results suggested a close association between the production of extracellular polysaccharides and the adaptability of *K. pneumoniae*. Additionally, our isolation of the two strains KP01 and KP02, both with intact *bcsA* and lacking mutations, implied the absence of a similar mechanism in the KL64-ST11 strain. In recent years, there has been a significant increase in the prevalence of KL64, a specific K-type of ST11, which consistently harbors a pLVPK-like virulence plasmid. Notably, KL64 exhibits greater virulence compared to KL47 (32). These findings indicate that the K-type, capsule production, and chain length of the *K. pneumoniae* capsule polysaccharide play important roles in biofilm formation, cell adhesion, and pathogenicity (9).

Many virulence genes, peg-344, *iroB*, and *iucA*; the plasmid-borne *rmpA* gene; and *rmpA2,* are also biomarkers of hypervirulent *K. pneumoniae* (16). RmpA serves as the principal regulator of mucoid bacteria, facilitating the production of capsule polysaccharides and enhancing bacterial pathogenicity (14). It is frequently found in the clonal group of hvKP strains and is closely associated with the mucoid phenotype (16). However, the reason why numerous *K. pneumoniae* strains expressing the complete *rmpA* gene exhibit low virulence and negative results on the string test has remained unclear in clinical practice. The most recent study by Liu et al. has identified five variants of the *rmpA* promoter, namely P_9T_ to P_13T_, exhibiting differences in their activity and geographic distribution. Nevertheless, there has been no investigation into the significance of these promoter variants in host niche colonization. Our study revealed that knocking out *rmpA* significantly reduced the mortality of ST11-KL64 bacteria in mice, which demonstrated that, compared with knocking out *iroB or iucA, rmpA* is crucial for pathogenicity. It was also found that strains carrying different types of *rmpA* promoters have differences in colonization of the host respiratory tract. Recently, the discovered *cps* regulatory factors *rmpC* and *rmpD* are closely related to bacterial hypermucoviscosity (HMV). The *rmpC* mutant exhibited a decrease in *cps* expression while still maintaining hypermucoviscous properties, suggesting that capsule formation and HMV may be separable traits. However, the small protein RmpD is crucial for HMV but does not affect the capsule structure (17). In addition, *rmpA*, *rmpD,* and *rmpC* are located within the operons regulated by RmpA (33). The phenotype associated with *rmpA* primarily arose from the activation of *rmpD*, resulting in the production of HMV, and the stimulation of *cps* expression by *rmpC* (17, 25). This finding suggested that the transcription level of the operon *rmpADC* can be controlled by the activity of the *rmpA* promoter, which, in turn, influences the virulence of *K. pneumoniae*. The acquisition of virulence plasmids is closely related to the spread of *K. pneumoniae*. Recently, Wilson et al. discovered a new virulence gene *iroP*, located in the salmochelin operon encoded by virulence plasmids. The IroP could respond to iron concentration regulated by the ferric uptake regulator (Fur), exerting opposite effects on the mucoid phenotype and expression of type 3 fimbriae (T3F) in *K. pneumoniae*. High iron conditions enhance T3F expression while suppressing the hypermucoid phenotype, thereby promoting biofilm formation and cell adhesion. Conversely, under iron-deficient conditions, a transcriptional switch occurs favoring capsule production and repression of T3F (34). Sequence alignment revealed that the *iroP* is present on both virulence plasmids of KP01 and KP02. However, the KP02 exhibited a low-viscosity phenotype due to the lack of *rmpA* promoter activity, which also leads to reduce the production of capsular polysaccharides. As a result, T3F may be likely to be exposed on the surface of the KP02 isolate. The *rmpADC* operon and *iroP* gene are regulated by Fur, but further research is needed to determine whether there is cross-regulation between *rmpA* and *iroP*.

**Fig 7:**
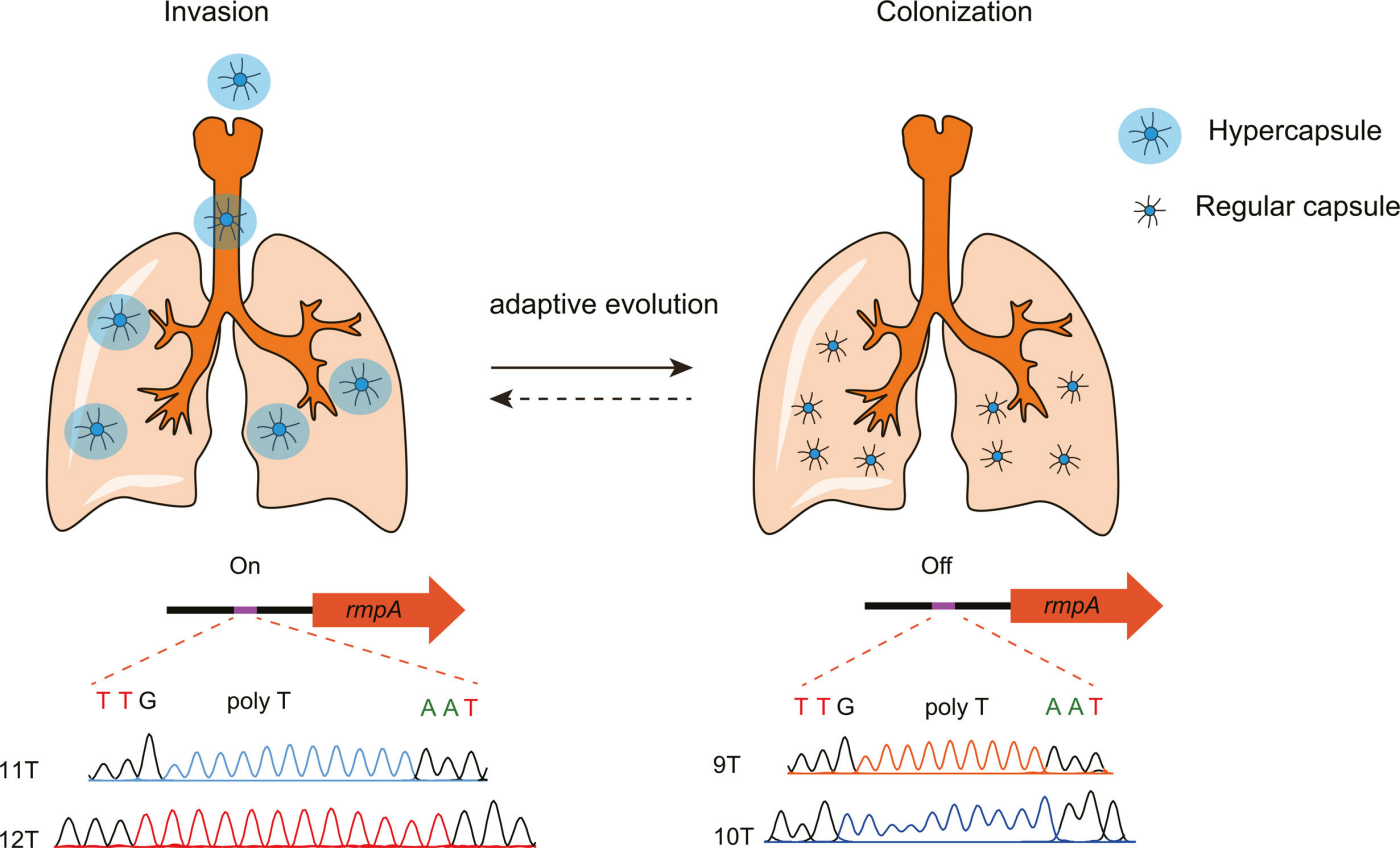
The evolutionary model of *K. pneumoniae* infection mediated by the *rmpA* promoter from invasion to colonization. Some *K. pneumoniae* strains exhibiting hypervirulence are characterized by the presence of the active P11T and P12T promoters of *rmpA*, which enable robust invasion and facilitate dissemination of the strain. Upon entering the host, the promoter of *rmpA* can undergo evolution, resulting in the emergence of P9T and P10T variants. This progress is mostly driven by the host immune system or antibiotic treatment. As a consequence, the hypermucoid disappeared, and the bacterial fimbriae became more exposed. Consequently, the strain’s capacity to establish colonization within the host was enhanced. Nevertheless, the potential for P9T and P10T to undergo evolutionary progression toward P11T and P12T could serve as strategic adaptations for bacteria. More research will be needed to determine the detailed process of phenotypic switching and the triggering factors involved.

In conclusion, we investigated the evolution of CRKP *in vivo* from two ST11-KL64 strains sharing similar genotypes yet exhibiting diverse phenotypic traits. Our findings indicated that virulence is regulated in some clinical *K. pneumoniae* strains through mutations in the *rmpA* promoter region during persistent infection. We found that there are five variants of promoters for *rmpA* in the NCBI database. The presence of P11T, P12T, and P13T can enhance the expression of *rmpA*, increase bacterial mucoidy, and cause the bacteria to become invasive and hypervirulent strains. P9T and P10T reduced the transcription level of *rmpA* and decreased capsule production. This may promote the exposure of type 3 fimbriae, thereby enhancing bacterial colonization. (Fig. 7). Collectively, our results indicated that the expression level of CPS is crucial for the virulence of ST11-KL64 and that the presence of the complete *rmpA* gene, which has an active promoter, can cause bacteria to become hypervirulent strains. This work demonstrated that the transition of nosocomial CRKP from hypervirulent strains to less harmful commensal bacteria is closely associated with mutations in the *rmpA* promoter region.

## MATERIALS AND METHODS

### String test

The string test was performed as previously described by Yao et al. (35). Briefly, bacteria were inoculated onto a Columbia blood plate overnight for cultivation. A positive result for the string test was that the viscous string length of a clone was greater than 5 mm, and a negative result for the string test was a length of less than 5 mm.

### Growth rate determination

To compare the different bacterial growth rates, strains KP01 and KP02 were transferred to cultivation at 37°C (180  rpm) in LB broth (36) (Table S3). The specific steps were as follows: the bacteria were shifted into fresh LB broth at 1:1,000 from the overnight cultures, and then the absorbance was measured at OD600 with Spectrophotometer (Spectrum Lab S32A) every hour interval for 24 h.

### Pulsed-field gel electrophoresis

The molecular typing was characterized by PFGE as previously reported (37). In short, the DNA of the standard strain *Salmonella* H9812 and tested strains were embedded by agarose plugs. The DNA plugs were digested by the restriction enzyme XbaI and separated by CHEF Mapper XA System (Bio-Rad, United States) in 0.5× Tris–borate–EDTA at 14°C for 18 h. The DNA fragments were visualized using Gel ComparII (Bio-Rad, United States) after ethidium bromide staining.

### Biofilm formation

After dilution overnight at a ratio of 1:1,000 to fresh LB medium, 200 µL was pipetted into each well of a 96-well polystyrene plate (38). Following a 48-h period of static incubation at 37°C, water was used to wash the wells, and the biofilm was fixed with 99% methanol. For 10 min, the biofilm was stained with 200 µL of 0.1% crystal violet. After removing the supernatant, the plates were washed three times with water. After dissolving crystal violet with 200 µL of 95% ethanol, the optical density at 570 nm was calculated. GraphPad Prism v.7.00 was used to examine statistical differences using a one-way ANOVA and Tukey’s post-hoc test.

### *Galleria mellonella* infection mode

We chose and bought 300 mg of *Galleria mellonella* larvae from Tianjin Huiyu Biotechnology Co., Ltd. in Tianjin, China. Before being used, they were recovered for 30 min at 37°C after being kept on woodchips in the dark at 4°C. Following the logarithmic phase culture of *K. pneumoniae*, the culture was washed with PBS and adjust the concentration to 10^7^ CFU/mL. Eight larvae per group were infected with the bacteria, and the hypervirulent strain of *K. pneumoniae* ATCC 43816 was used as the control strain to report the larvae’s survival rate after 72 h (1). Every experiment was carried out three times.

### Whole-genome sequence and bioinformatics analysis

As previously mentioned, the Illumina HiSeq platform was utilized to extract and sequence the genomic DNA of the aforementioned strains (MEIGE,Guang Zhou). The Nanopore platform (Oxford Nanopore Technologies, Oxford, UK) was utilized for the long-read library creation of KP01 and KP02. To assemble the reads *de novo* for KP01 and KP02, the HGAP workflow found in SMRT Analysis v2.3.0 was employed. Plasmids were identified for the resultant large contigs by BLASTP/BLASTN searches, and RAST v2.0 (https://rast.nmpdr.org) was used for annotation (39). PlasmidFinder v2.1 (https://cge.food.dtu.dk/services/PlasmidFinder) was utilized to investigate plasmid incompatibility, whereas ResFinder v.4.1 (https://cge.food.dtu.dk/services/ResFinder) was employed to identify resistance loci. Utilizing ISfinder (https://www-is.biotoul.fr/index.php) to predict insertion sequences and oriTfinder (https://bioinfo-mml.sjtu.edu.cn/oriTfinder) to identify conjugation elements, it is possible to predict plasmid propensity for conjugation. The BRIG program was used to create circular plasmid maps (40).

### Mouse infection model

Using a mouse bacteremia model to assess the virulence of different *K. pneumoniae* strains (41, 42). Female CD-1 mice (average ~20 g, 6 weeks) were purchased from the HANGZHOU QIZHEN LABORATORY ANIMAL TECHNOLOGY CO., LTD (Hangzhou, China) and allowed to provide food and water throughout the entire research process. Divide eight mice into a group. Each group of mice was injected intraperitoneally or intranasally to evaluate the pathogenicity of different *Klebsiella pneumoniae* strains. The amount of *K. pneumoniae* injected per mouse was 10^7^ CFU. Observe and record the mortality rate of experimental mice 120 h after infection. The control strain was selected as the highly virulent strain ATCC 43816. The animal experiments were conducted independently three times, with one result chosen as representative. Generate a survival curve for GraphPad Prism v.7.00. Statistical analysis was performed using logarithmic rank (Mantel-Cox) for Prism v.7.00 recommended tests.

### Bacterial cell adhesion

HeLa and A549 cells were grown in DMEM with 10% FBS and 1× penicillin-streptomycin added. Every mammalian cell was kept at 37°C with 5% CO_2_ in a humidified incubator. On 24 well plates, the different cells were cultured at a density of 0.25 × 10^6^. The cells were infected with bacteria at a multiplicity of infection (MOI) of 10, and they were then incubated for 30 min at 37°C. The cells were lysed using 0.1%–0.25% Triton X-100 (Sigma) following three rounds of washing in 1× PBS. Overnight incubation at 37°C was performed on lysogeny broth agar (LBA) plates containing the appropriate dilutions (10, 43).

### Gene knockout and mutation construction

As mentioned earlier, gene knockout of *Klebsiella pneumoniae* was performed using the CRISPR/Cas9 editing system (44, 45). To knock out the *rmpA* virulence genes in KP01 and KP02, 500 bp upstream and downstream of the *rmpA* gene were amplified and used as donor DNA for homologous recombination fragments. From *E. coli* DH5α, the pSGKP-Apr-*rmpA* spacer plasmid extracted from was then electroporated with donor DNA into pCasKP-Rif carrying KP01 and KP02. Verify the correct mutant through PCR and Sanger sequencing. The method for a point mutation in the *rmpA* promoter region is the same as above. To eliminate the virulence plasmid, the 20 bp spacer sequence from *rmpA* was cloned into pSGKP-Apr to construct the pSGKP-Apr-*rmpA* spacer plasmid and electroporated into pCasKP-Rif carrying *Klebsiella pneumoniae* isolates without donor DNA. All eliminated virulence plasmid clones were confirmed by PCR. The plasmids and primers used are listed in Tables S5 and S6.

### RNA isolation, purification, and library preparation for sequencing

Incubate KP01 and KP02 strains overnight in LB broth at 37°C and 180 rpm and transfer them to 20 mL of fresh LB in a 1:100 ratio. When the culture reaches the exponential growth stage (OD_600_ = 0.6–0.8), cells are collected by centrifugation at 12,000 rpm for 10 min at 4°C. According to the manufacturer’s instructions, total RNA was extracted using TRIzol reagents (Invitrogen, Waltham, MA, USA), and RNA samples were purified using RNeasy mini kits (Qiagen, Germantown, MD, USA). The quality of RNA was determined by running 1.2% agarose gel, and the concentration of RNA was further detected by NanoDrop 2000 spectrophotometer (Thermo Fisher, Waltham, MA, USA) (46). RNA library preparation and high-throughput sequencing were carried out by MAGIGENE (Guangzhou, China). Use HISAT2 (v2.2.0) (http://daehwankimlab.github.io/hisat2/) (47) to map the original sequencing readings to the KP01 genome reference (PRJNA1039950).

### Quantitative real-time qPCR

The extracted RNA was reverse transcribed into cDNA using the EvoM-MLV RT Mix Kit with gDNA Clean for qPCR ver.2 (Accurate Biology,China). Use Novostart SYBR qPCR Supermix Kit (Novoprotein) for real-time qPCR. Standardize relative RNA transcripts relative to wild-type controls using the 2^−ΔΔCt^ threshold cycling method, with *rpoB* as the internal reference control (26). All qPCR primers are listed in Table S6.

## Data Availability

The Nanopore sequencing data of the *K. pneumoniae* isolate KP01 and KP02 were deposited in NCBI under accession number PRJNA1039950. The RNA-seq data used in this work are available in the NCBI SRA database under the accession code PRJNA1041962. Other data are presented within the paper and supplemental material.

## SUPPLEMENTAL MATERIAL

Supplemental figures msystems.01363-23-s0001.docx Fig. S1 to S4.

Supplemental tables msystems.01363-23-s0002.xlsx Tables S1 to S6.

ASM does not own the copyrights to Supplemental Material that may be linked to, or accessed through, an article. The authors have granted ASM a non-exclusive, world-wide license to publish the Supplemental Material files. Please contact the corresponding author directly for reuse.
