# Supplementary material for: Adaptive attenuation of virulence in hypervirulent carbapenem-resistant Klebsiella pneumoniae
Source: mSystems. 2024 May 16;9(6):e01363-23. doi: 10.1128/msystems.01363-23 (PMC11237801; doi:10.1128/msystems.01363-23)
Supplement: Supplemental figures — Fig. S1 to S4. [file msystems.01363-23-s0001.docx]

**Supplementary figures**


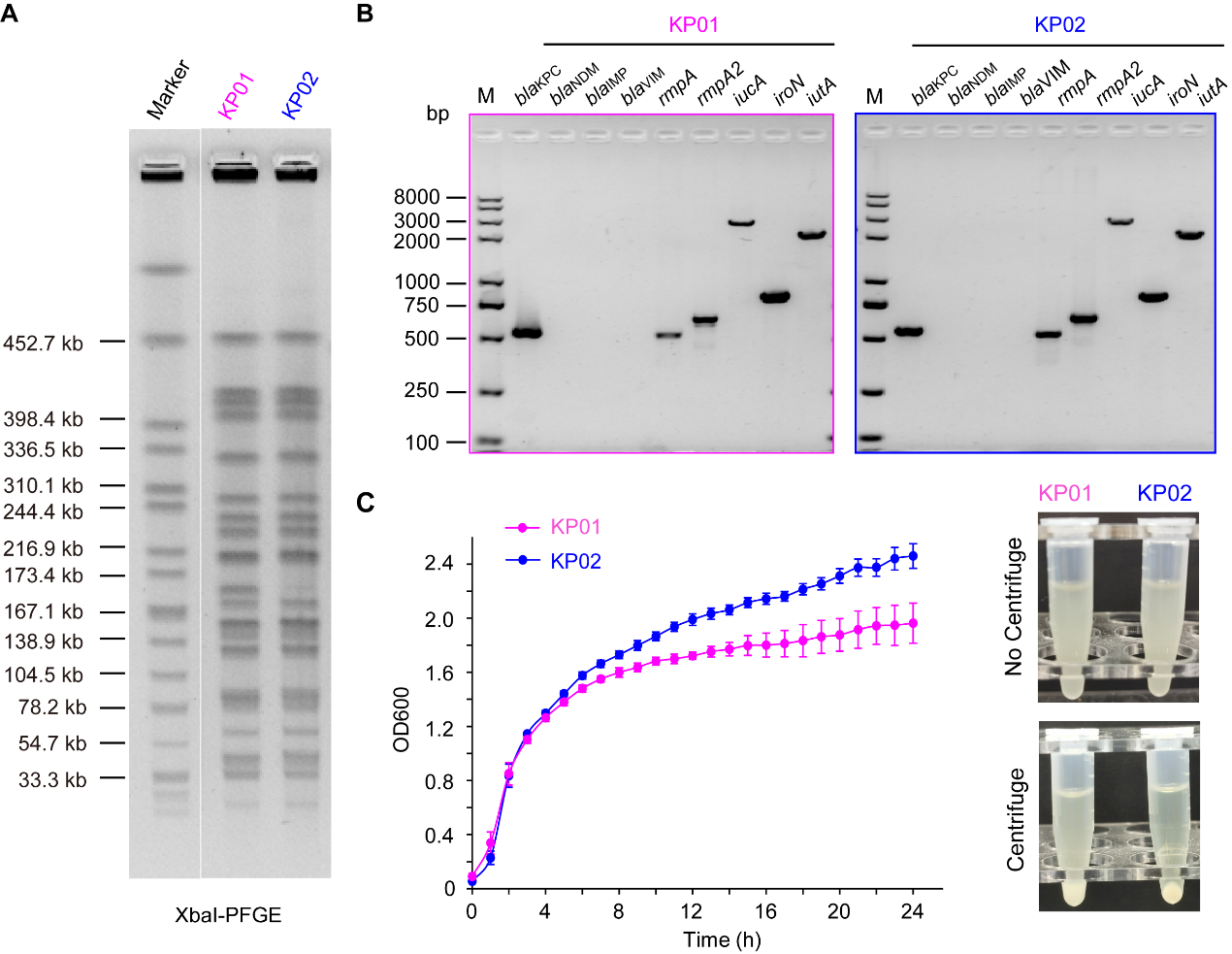


**Fig.S1** The characterization of KP01 and KP02 isolated from the same patient

**A**. Analysis of the genomic profiles of KP01 and KP02 by XbaI-PFGE

**B**. Detection of carbapenem resistance genes and virulence genes in *K. pneumoniae* by PCR

**C**. Comparison of growth rates and mucoid sedimentation of KP01 and KP02


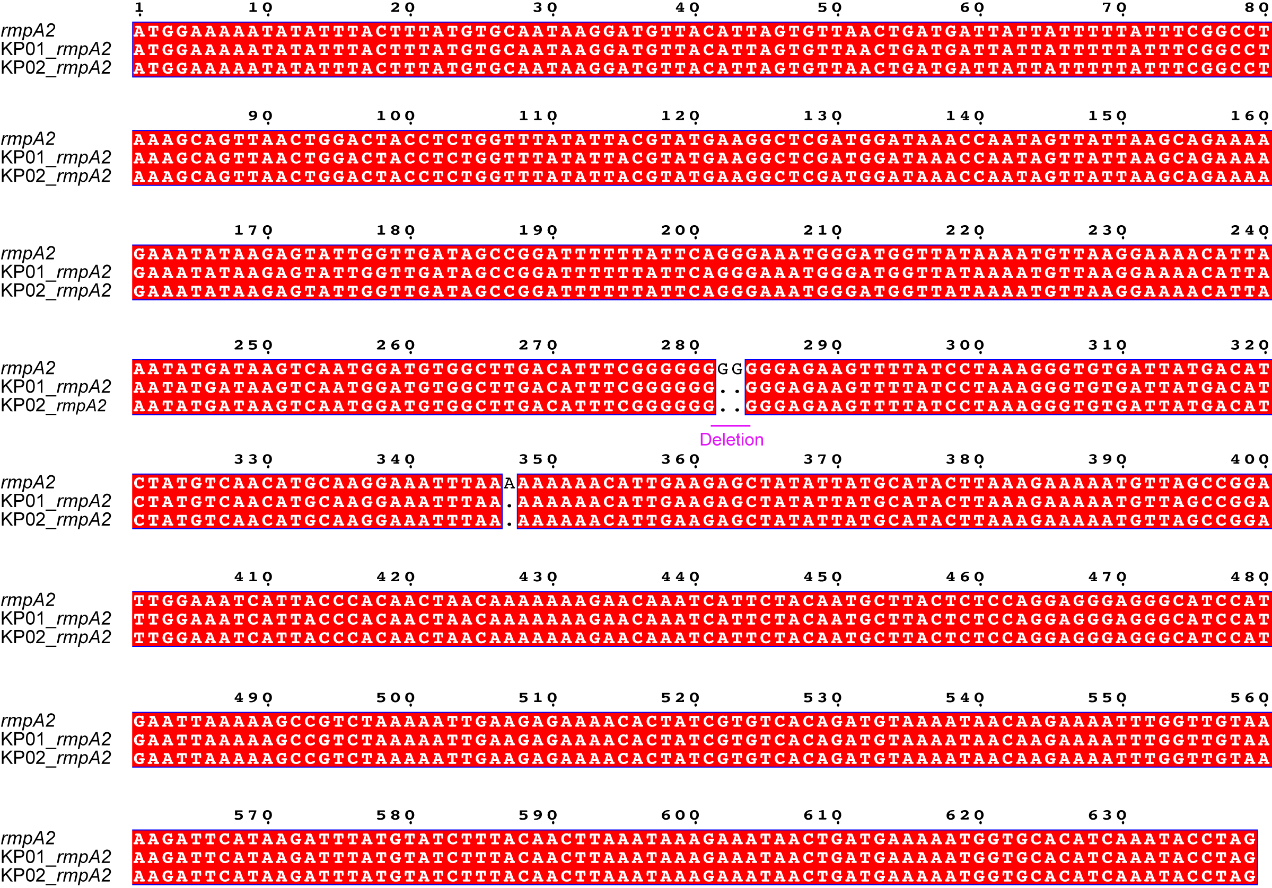
**Fig S2**. Comparison between the presence of deletion mutations in the *rmpA*2 gene in virulence plasmid of KP01 and KP02.


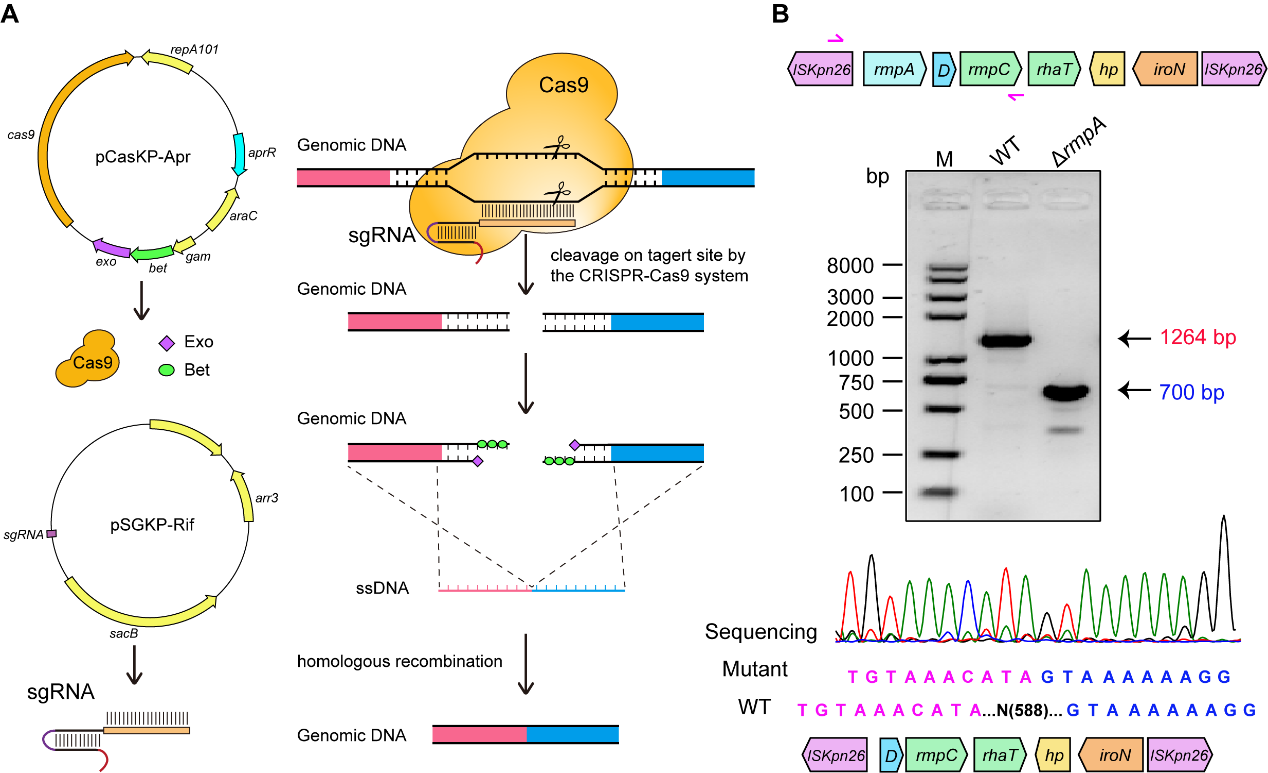


**Fig S3**. Genome editing in *K. pneumoniae* using a CRISPR/Cas9 system

**A**. Scheme for knock-out of *rmpA* based on the CRISPR/Cas9 system

**B**. PCR was used to validate the *rmpA* deletion mutants. DNA sequencing-based analyses for the wild-type *rmpA* and its deletion mutant Δ*rmpA*


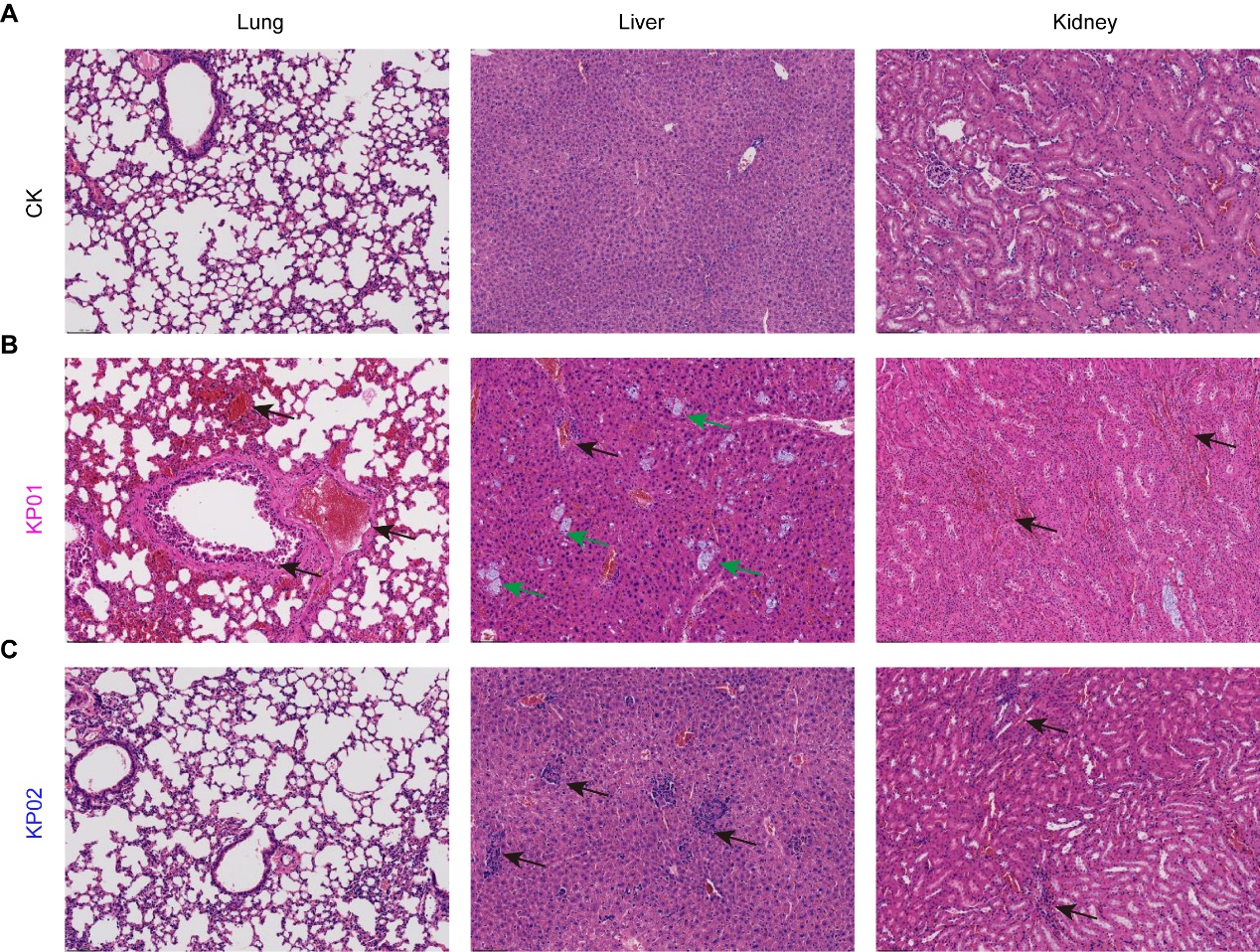


**Fig S4.** Pathological section of the different tissue from mice infected with KP01 and KP02

**A**. Hematoxylin-eosin (HE) staining images for the tissues of lung, liver and kidneys from CD-1 mice infected with the PBS, the group as the negative control.

**B**. mice tissues following the challenge with KP01, a ST11-KL64 hvKP strain (carried a P11T *rmpA* virulence plasmid)

**C.** Pathological sections of the mouse lung, liver and kidneys from the mouse infected with the KP02, a ST11-KL64 lvKP strain.

In brief, the congestion sites are indicated with arrows, and the abscess regions are highlighted with green arrows.

**Supplementary references**

Mina SA, Zhu G, Fanian M, Chen S, Yang G. Exploring reduced macrophage cell toxicity of hypervirulent Klebsiella pneumoniae compared to classical *Klebsiella pneumoniae*. Microbiol Res. 2024 Jan;278:127515

Zhang F, Meng Y, Xu L, Tian Y, Lu H, Xie J, Ma R, Li M, Li B. KbvR mutant of Klebsiella pneumoniae affects the synthesis of type 1 fimbriae and provides protection to mice as a live attenuated vaccine. Vet Res. 2022 Nov 26;53(1):97

Zhang Y, Jin L, Ouyang P, Wang Q, Wang R, Wang J, Gao H, Wang X, Wang H; China Carbapenem-Resistant Enterobacteriaceae (CRE) Network. Evolution of hypervirulence in carbapenem-resistant *Klebsiella pneumoniae* in China: a multicentre, molecular epidemiological analysis. J Antimicrob Chemother. 2020 Feb 1;75(2):327-336.
